# Relational representations in classification: comparing similarity and structure-based approaches

**DOI:** 10.3389/fpsyg.2026.1942038

**Published:** 2026-10-12

**Authors:** Jongwan Kim, Kimin Eom

**Affiliations:** 1 Department of Psychology, Jeonbuk National University, Jeonju-si, Jeonbuk, Republic of Korea; 2 Research School of Management, The Australian National University, Canberra, ACT, Australia

**Keywords:** classification, feature representation, multidimensional scaling, relational representation, structure-based classification

## Abstract

Classification has become an important analytical tool in psychology, yet methodological research has focused primarily on improving learning algorithms, while the representation of psychological information has received comparatively less attention. This study examines how three representational strategies affect classification performance: a similarity-based representation derived from multidimensional scaling, an intra-stimulus relational representation (structure-based classification, SBC), and a conventional feature-based representation. Across three simulation studies and two empirical datasets from emotion perception, we evaluated these representations under a common classification framework. When class structure was defined purely by the location of stimuli in a latent space, similarity-based and feature-based representations performed comparably, and both significantly outperformed the relational representation. Under the primary linear-SVM analyses, the relational representation became more effective at low separation when classes differed in dispersion, but declined disproportionately as task-irrelevant dimensions increased. Nonlinear RBF-SVM robustness analyses showed that both effects were partly classifier-dependent: dispersion-related information could also be recovered from feature- and similarity-based representations, and the dimensionality-related SBC decline was substantially attenuated. Empirical applications showed a similarly mixed pattern, with the relative performance of each representation varying across datasets. These findings indicate that no single representation is universally optimal; rather, each representation is differentially sensitive to specific statistical properties of class structure, including location, dispersion, and dimensionality. Representational choice should therefore be treated as a substantive modeling decision in psychological classification rather than a routine preprocessing step.

## 1 Introduction

Psychological research often represents stimuli, responses, or individuals as vectors of feature values, that is, as sets of measured scores across multiple dimensions, such as Likert-type ratings. These feature-based representations are then used as inputs to classification models that aim to predict categories, affective states, or perceptual judgments. Although this approach is straightforward and widely applicable, it assumes that absolute rating values provide a stable basis for comparison across individuals and stimuli. This assumption is not always warranted. In self-report research, responses are shaped by scale-use tendencies, response styles, reference-group effects, and contextual influences, all of which can obscure psychologically meaningful information when raw scores are analyzed directly (Friedman and Amoo, 1999; Schwarz, 1999; Heine et al., 2002). More broadly, psychological meaning may reside not only in individual feature values but also in relationships among responses. In the present study, we use the term representation in a restricted methodological sense, referring primarily to multivariate behavioral or psychophysical representations derived from feature ratings or inter-stimulus similarity structure, rather than to neural representations directly.

Recent methodological work has increasingly emphasized relational representations as an alternative to purely feature-based approaches. One example is the structure-based classification (SBC) framework, which represents each stimulus in terms of relationships among rating dimensions rather than raw rating scores (Kim, 2026). By encoding pairwise relations among features, SBC captures intra-stimulus relational structure and can improve classification when class-relevant information is embedded in inter-dimensional patterns rather than in marginal feature values. A related perspective appears in representational similarity analysis (RSA), where relationships among stimuli are modeled geometrically using similarity structures rather than independent feature vectors (Kriegeskorte et al., 2008; Diedrichsen and Kriegeskorte, 2017). Together, these approaches suggest that classification performance may depend not only on the features used to represent stimuli, but also on the level at which relational information is encoded.

For example, consider an affective-rating study in which a researcher seeks to classify sensory stimuli into different affective categories based on ratings such as enjoyment, calmness, tension, and unpleasantness. A feature-based classifier would use the absolute rating levels, whereas SBC would ask whether the categories differ in the pattern of differences among ratings within each stimulus, for example, whether the relative configuration of enjoyment, calmness, tension, and unpleasantness remains informative even when absolute response levels vary. A similarity-based representation would instead ask whether stimuli from the same category occupy nearby positions in a shared space defined by their overall rating profiles.

However, the relational structure captured by SBC is defined within stimuli, focusing on relationships among features for a given observation. In many classification settings, relevant structure may instead arise between stimuli. Similarity judgments and perceptual comparison tasks, for example, represent stimuli in terms of their relations to other stimuli rather than in terms of independent feature values. This perspective has a long tradition in multidimensional scaling (MDS), where pairwise similarity or dissimilarity data are used to recover a lower-dimensional perceptual map in which stimuli are positioned according to their relative similarities (Torgerson, 1958; Kruskal and Wish, 1978). When class distinctions are reflected in the overall pattern of how stimuli are positioned relative to one another, representations derived from similarity structure may provide informative features for classification.

This distinction suggests that relational representations in classification can operate at different levels. SBC captures intra-stimulus structure by modeling relationships among features within individual stimuli. In contrast, similarity-based representations capture inter-stimulus structure by representing stimuli in terms of their positions in a shared perceptual space. These approaches, therefore, rely on different assumptions about where class-relevant information is encoded. When classification depends primarily on global similarity structure, inter-stimulus representations may be advantageous. In contrast, when information is embedded in relationships among features within stimuli, intra-stimulus representations may provide more effective classification features.

The present study examines representational choice as a methodological issue in psychological classification. Specifically, we compare three representational strategies: an inter-stimulus similarity representation derived from multidimensional scaling, an intra-stimulus relational representation implemented through the SBC framework, and a conventional feature-based representation. Using simulated and empirical datasets, we investigate how these representational choices influence classification performance across data structures that differ in class location, within-class dispersion, and dimensionality. Rather than proposing a universally superior method, we aim to identify the conditions under which different representational strategies are most appropriate for psychological classification. Conceptually, feature-based representations are most relevant when category information is expected to reside in absolute feature levels, intra-stimulus relational representations when it resides in relations among features within an observation, and inter-stimulus representations when categories are organized by relative positions within a shared similarity space.

The methodological question addressed in this study extends beyond the specific datasets examined here. Classification and predictive modeling are increasingly used in psychology to decode perceptual or cognitive states from multivariate patterns, to predict clinically relevant outcomes, and more broadly to evaluate out-of-sample prediction of psychological variables (Pereira et al., 2009; Dwyer et al., 2018; Yarkoni and Westfall, 2017). However, these applications commonly assume that the choice of representation is secondary to the choice of classifier. The present study challenges this assumption by demonstrating that classification performance depends on whether the selected representation matches the structure through which psychologically meaningful information is organized.

To evaluate this proposition, we conducted three simulation studies in which class-relevant information was systematically manipulated along different statistical properties: the location of class means, the dispersion of within-class variability, and the dimensionality of task-irrelevant noise. These simulations were complemented by empirical analyses of affective datasets involving sensory stimuli. Together, these analyses illustrate both the potential advantages and the boundary conditions of alternative representational strategies for psychological classification. Although the empirical examples focus on affective data, the representational distinction examined here may be relevant more broadly whenever psychological observations can be characterized through feature values or similarity relations.

## 2 Representational framework

Let $X\in {\mathbb{R}}^{n\times p}$ denote a dataset consisting of *n* stimuli described by *p* features or rating dimensions. Traditional classification approaches represent each stimulus directly using its feature vector $x_{i}\in {\mathbb{R}}^{p}$ .

To provide a self-contained comparison, we briefly summarize the SBC procedure here. For a stimulus described by p feature dimensions, SBC constructs a relational feature vector from all pairwise comparisons among the *p* dimensions (e.g., differences or ratios between dimension pairs). This relational vector is then used directly as input to the classifier, in place of the original *p*-dimensional feature vector. Unlike the similarity-based representation developed in the present study, which encodes relations between stimuli, SBC encodes relations within a single stimulus’ own feature profile (Kim, 2026).

In contrast, similarity-based representations encode each stimulus in terms of its relationships to other stimuli. Given a pairwise dissimilarity matrix $D\in {\mathbb{R}}^{n\times n}$ , where D*_ij_* represents the dissimilarity between stimuli *i* and *j*, two-dimensional MDS is used to obtain a low-dimensional embedding of the stimuli (Torgerson, 1958; Kruskal and Wish, 1978). Specifically, MDS produces a coordinate matrix


$$Y={\left[y_{1},y_{2},\ldots ,y_{n}\right]}^{⊤}\in {\mathbb{R}}^{n\times d},$$

where each stimulus is represented by a d-dimensional coordinate vector y*_i_*, and pairwise Euclidean distances in the embedded space approximate the original dissimilarities in D.

These coordinate vectors are then used as inputs for classification. Unlike SBC, which represents each stimulus by relationships among features within the stimulus itself, the present approach represents stimuli by their positions within a shared similarity structure. The resulting representation, therefore, captures inter-stimulus relational information rather than intra-stimulus feature structure. Importantly, similarity information can be obtained either directly from similarity judgments or indirectly from distances among multivariate feature profiles. In the present study, the latter approach was used; thus, the similarity-based representation remains dependent on the dimensions selected for measurement, although those measurements are subsequently encoded in terms of inter-stimulus relations rather than absolute feature values.

## 3 Simulation study

To evaluate the proposed representational framework, we conducted three simulation studies comparing similarity-based classification against SBC and traditional feature-based classification. Simulation 1 established a baseline condition in which the two classes differed only in their mean locations while sharing the same covariance structure. Simulation 2 examined a heterogeneous-dispersion condition in which the two classes additionally differed in covariance, to test whether representations could exploit dispersion-related information beyond mean separation. Simulation 3 examined classification performance as the number of irrelevant (noise) dimensions increased, while holding class separation and covariance structure constant, to assess each representation’s robustness to increasing dimensionality. All three simulations used the same linear support vector machine classifier and cross-validation procedure (see Classification procedure below) to isolate differences attributable to representation.

### 3.1 Classification procedure

Three representational approaches were compared across all simulations: (a) similarity-based classification, (b) SBC, and (c) traditional feature-based classification. All three representations were classified in the primary analyses using the same linear support vector machine, implemented with default hyperparameters and no additional tuning (MATLAB *fitcsvm*, linear kernel). A linear SVM was selected to provide a common, low-complexity decision rule across representations, thereby making performance differences more directly interpretable in terms of representational structure. This choice was not intended to imply that linear SVMs are uniquely optimal for psychological data; rather, SVMs are well-established tools for multivariate classification in psychological and neuroimaging research, and the linear formulation provides a deliberately constrained readout when the primary goal is to compare representations rather than to optimize predictive performance (Pereira et al., 2009). To assess whether the observed effects depended on this linear decision rule, we additionally repeated all three simulations in a matched robustness analysis using an RBF-kernel SVM (*KernelScale = ‘auto’, BoxConstraint = 1, with no hyperparameter optimization*). Within each simulated dataset in this robustness analysis, the same 10-fold cross-validation partition was used across all three representations and both classifiers. For Simulations 1 and 2, classification accuracy was estimated using 10-fold cross-validation, repeated across 100 independently generated datasets per condition. This number of iterations was chosen as a pragmatic balance between obtaining repeated Monte Carlo estimates of performance and the computational demands of repeatedly constructing the three representations and fitting the classifiers. Simulation 3 used 50 iterations per condition because the number of SBC features increases quadratically with dimensionality, substantially increasing computational cost at the higher-dimensional conditions. For the similarity-based approach, pairwise Euclidean distances among stimuli were computed and transformed into a two-dimensional embedding (*d* = 2). For SBC, each stimulus was represented using pairwise relational features constructed from all pairwise combinations of the latent feature dimensions (Kim, 2026). Traditional classification used the original feature vectors directly, without relational transformation. These shared procedures were applied identically across the three simulations described below, so that any differences in classification performance could be attributed to the underlying data structure rather than to differences in classifier implementation. To illustrate how the same underlying observations are transformed by each representational approach, representative examples of the feature-based, SBC, and similarity-based representations from each simulation are shown in Supplementary Figure S1.

### 3.2 Simulation 1: baseline separation design

Two classes of stimuli were constructed in a latent four-dimensional space. For each condition, *n* = 200 stimuli were generated, with 100 assigned to each class. The sample size of 200 was chosen to provide a reasonably sized classification problem for 10-fold cross-validation while keeping the repeated distance, MDS, and SBC transformations computationally tractable. A four-dimensional space was chosen as a simple multivariate baseline that retained a manageable number of relational features for SBC while allowing dimensionality to be increased systematically in Simulation 3. The simulations were restricted to two classes to isolate representational effects under a controlled and readily interpretable decision structure, rather than to model the additional complexity introduced by multiclass classification. These parameter values were therefore intended as controlled design choices rather than as psychologically privileged constants. Both classes were sampled from multivariate normal distributions with identical covariance matrices (0.6 × I₄), differing only in their means: Class A was centered at the origin, and Class B was displaced along the first latent dimension by an amount controlling class separation. The covariance magnitude of 0.6 was selected to provide moderate within-class variability relative to the range of mean separations, allowing performance to be examined from near-chance conditions through progressively stronger class separation without immediate ceiling performance. This equal-covariance design isolates the effect of pure mean separation, independent of any dispersion-based cues. The equal-covariance assumption was therefore used as a controlled baseline rather than as a claim that psychological categories generally exhibit identical variability; Simulation 2 explicitly relaxed this assumption by allowing the classes to differ in dispersion. The distance between class centroids ranged from 0 to 5.0 in increments of 0.5 (11 levels, including a null-separation baseline). Pairwise Euclidean distances among stimuli were computed to form a dissimilarity matrix *D*, which was submitted to two-dimensional MDS to obtain a two-dimensional coordinate representation (*d* = 2) for the similarity-based approach. All three representations (similarity-based, SBC, and traditional feature-based) were derived from this same underlying latent structure and classified using the procedure described above.

Figure 1 (left panel) summarizes classification accuracy across separation conditions for the equal-covariance design. At zero separation, all three approaches performed near chance level (similarity-based classification: 49.09%; SBC: 49.96%; Traditional: 49.25%), and the difference between similarity-based classification and SBC was not significant, *t*(99) = −1.47, *p* = 0.144. As separation increased, similarity-based classification accuracy rose sharply and significantly exceeded SBC’s at every separation level from 0.5 to 5.0 (all *p*s < 0.0001; Table 1), with the largest advantage observed at intermediate separation (Separation = 1.0: similarity-based classification = 73.56%, SBC = 61.02%, mean difference = 12.55 percentage points, *t*(99) = 31.30, *p* < 0.0001) and a narrowing gap toward the ceiling (Separation = 5.0: similarity-based classification = 99.90%, SBC = 99.30%, mean difference = 0.61 percentage points, *t*(99) = 8.72, *p* < 0.0001). By contrast, similarity-based classification accuracy closely tracked the traditional feature-based approach across all separation levels (e.g., Separation = 1.0: similarity-based classification = 73.56%, Traditional = 73.51%), with no evidence of a systematic advantage for either representation in this design.

**Figure 1:**
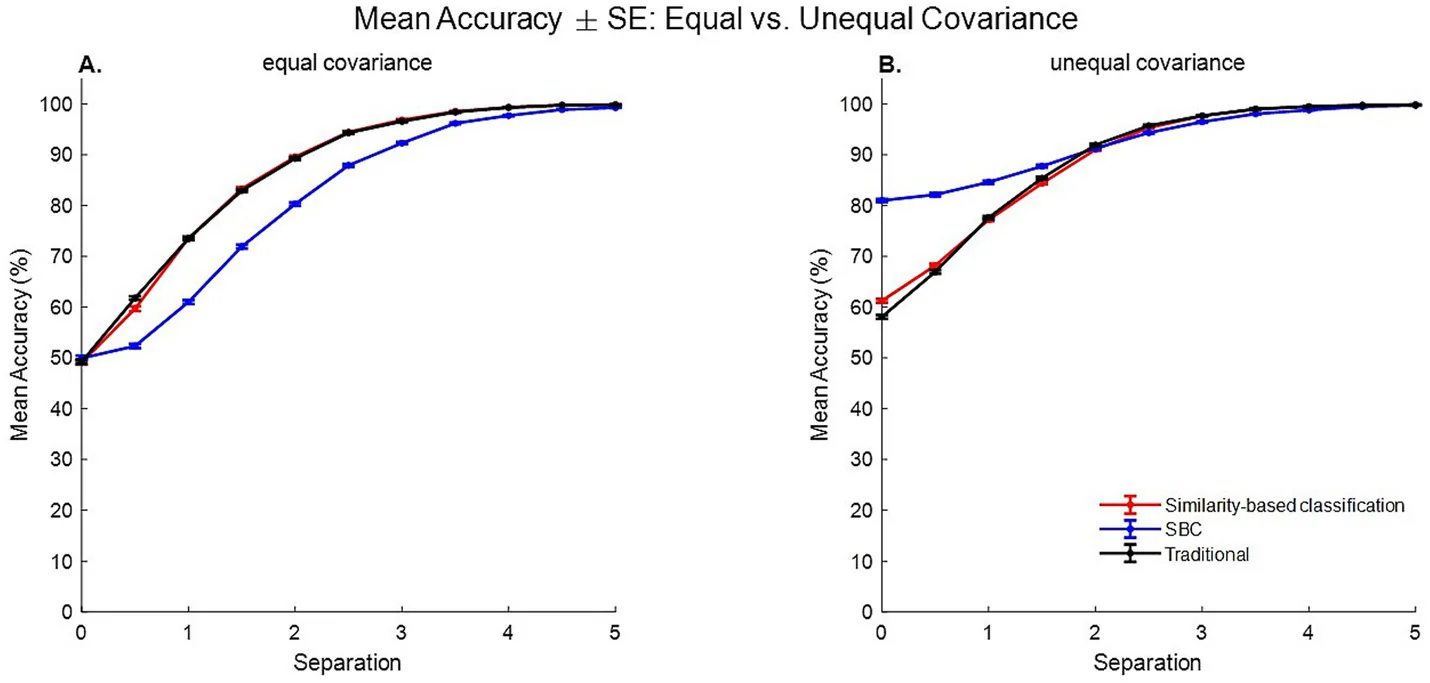
Mean classification accuracy (± standard error) across class separation levels for similarity-based classification, SBC, and traditional feature-based classification, under equal-covariance (Panel **A**; Simulation 1) and unequal-covariance (Panel **B**; Simulation 2) generative designs. Error bars represent ±1 SE across 100 iterations per condition. Separation values reflect the Euclidean distance between class centroids in the latent space.

**Table 1:** Similarity-based classification vs. SBC accuracy comparisons across separation levels (Simulations 1 and 2).

Separation	Equal covariance			Unequal covariance		
	diff (similarity-based classification−SBC)	*t*	*p*	diff (similarity-based classification−SBC)	*t*	*p*
0.0	−0.009	−1.47	0.144	−0.198	−49.65	<0.0001
0.5	0.074	12.94	<0.0001	−0.139	−38.04	<0.0001
1.0	0.126	31.30	<0.0001	−0.074	−21.16	<0.0001
1.5	0.114	38.67	<0.0001	−0.033	−12.48	<0.0001
2.0	0.093	28.75	<0.0001	−0.002	−1.06	0.293
2.5	0.066	25.64	<0.0001	0.010	5.00	<0.0001
3.0	0.045	22.53	<0.0001	0.012	8.31	<0.0001
3.5	0.024	15.01	<0.0001	0.009	9.05	<0.0001
4.0	0.017	13.13	<0.0001	0.007	8.11	<0.0001
4.5	0.009	11.20	<0.0001	0.003	5.25	<0.0001
5.0	0.006	8.72	<0.0001	0.001	2.89	0.005

These results indicate that under a purely location-based separation structure, similarity-based representations preserve as much class-relevant information as the original feature vectors. In contrast, relational (SBC) representations discard a substantial portion of this information, because pairwise feature differences are largely uninformative when class separation is expressed through absolute location rather than internal feature relationships.

### 3.3 Simulation 2: heterogeneous dispersion

Simulation 2 used the same generative structure as Simulation 1, except that the two classes differed in covariance in addition to mean location: Class A was sampled with covariance 0.2 × I₄, and Class B with covariance 1.0 × I₄. These values were chosen to create a clear contrast in within-class dispersion while retaining isotropic covariance within each class, thereby varying dispersion magnitude without introducing differences in covariance orientation or inter-dimensional correlations. This design introduces a second source of class-relevant information, differences in within-class dispersion, that coexists with the mean-separation signal used in Simulation 1.

Figure 1 (right panel) summarizes accuracy under the heterogeneous-dispersion design. Unlike Simulation 1, SBC significantly outperformed similarity-based classification at low separation levels (Separation = 0: similarity-based classification = 61.25%, SBC = 81.00%, mean difference = −19.75 percentage points, *t*(99) = −49.65, *p* < 0.0001; Separation = 1.5: mean difference = −3.34 percentage points, *t*(99) = −12.48, *p* < 0.0001), reflecting SBC’s sensitivity to the dispersion difference between classes even when mean separation was minimal or absent. This advantage reversed at higher separation levels, where similarity-based classification modestly but significantly outperformed SBC (Separation ≥ 2.5, all *p*s < 0.0001; Table 1), consistent with the pattern observed in Simulation 1 once the mean-separation signal came to dominate.

Under the primary linear-SVM analysis, SBC’s relational features were particularly informative when class-relevant information included within-stimulus dispersion cues, whereas feature-based and similarity-based representations were more effective when class information was expressed primarily through location differences. As shown by the nonlinear robustness analysis below, however, the apparent SBC advantage for dispersion-related information depended in part on classifier flexibility.

### 3.4 Simulation 3: high-dimensional noise design

Simulation 3 examined the robustness of each representation as the number of task-irrelevant (noise) dimensions increased. Class separation was fixed at an intermediate value (separation = 2.0, corresponding to the steepest region of the accuracy curves in Simulation 1), and both classes shared identical covariance (1.0 × I) across all dimensions, following the equal-covariance design of Simulation 1. Unit variance was used so that each added noise dimension had the same standardized variability, ensuring that the dimensionality manipulation changed the number rather than the scale of irrelevant dimensions. The total number of latent dimensions was systematically varied (4, 6, 10, 15, 20, and 30), with the class-separation signal confined to the first dimension in every condition; all additional dimensions were uncorrelated noise. Because the number of pairwise SBC features grows quadratically with dimensionality, 50 iterations per condition were used (rather than 100, as in Simulations 1 and 2) to keep computation tractable.

Figure 2 shows the mean classification accuracy as a function of total dimensionality. Similarity-based classification accuracy remained stable across the dimensionality range (80.66–83.79%), as did the traditional approach (79.18–82.89%). Similarity-based classification did not consistently outperform the traditional approach: the two representations differed significantly at some dimensionalities (10 dimensions: mean difference = 0.90 percentage points, *t*(49) = 2.99, *p* = 0.004; 15 dimensions: mean difference = 0.75 percentage points, *t*(49) = 2.38, *p* = 0.021; 30 dimensions: mean difference = 1.48 percentage points, *t*(49) = 3.31, *p* = 0.002) but not at others (4, 6, and 20 dimensions: all *p*s > 0.13; Table 2), and the significant differences were small in magnitude (< 1.5 percentage points).

**Figure 2:**
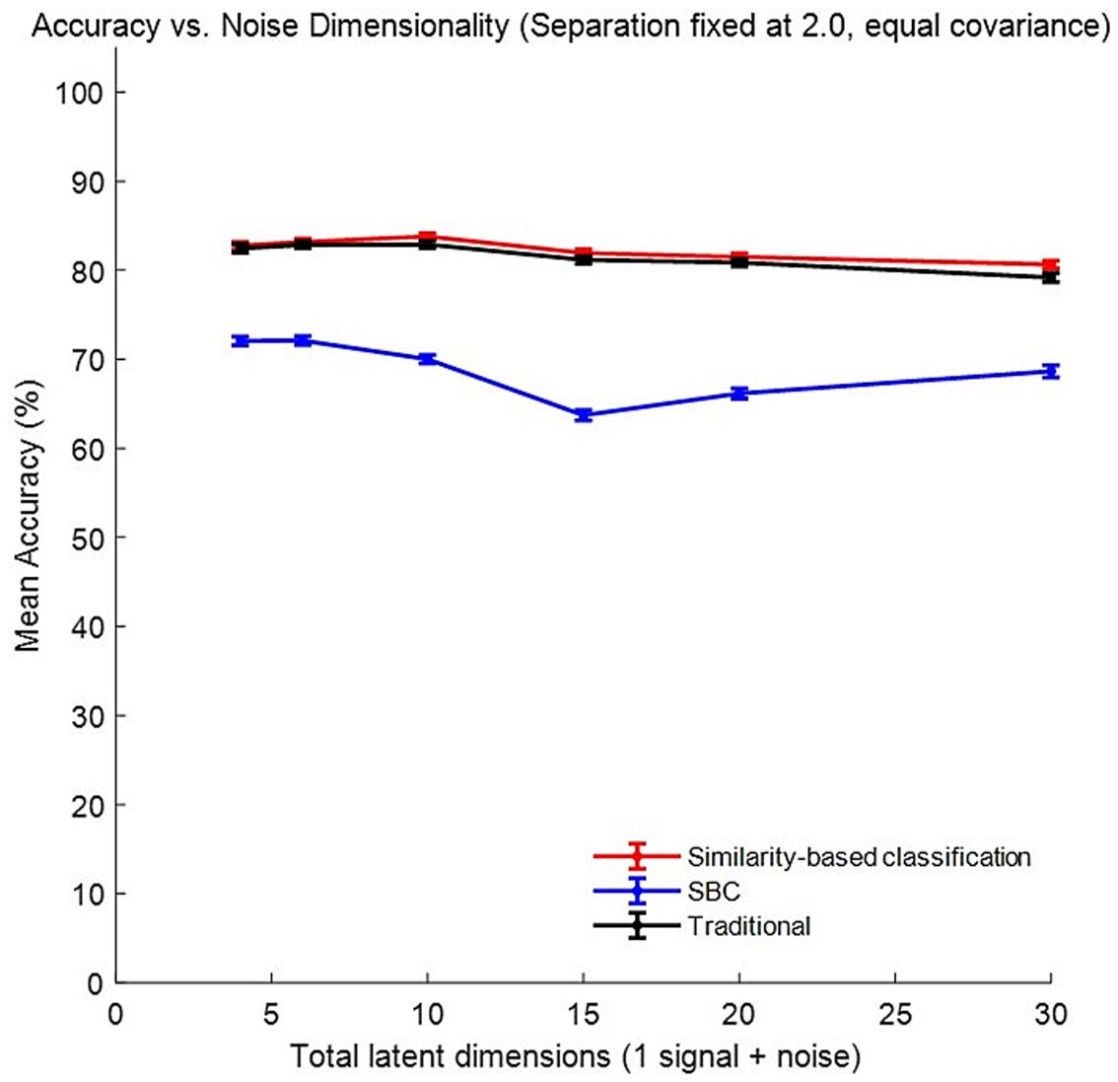
Mean classification accuracy (± standard error) as a function of total latent dimensionality for similarity-based classification, SBC, and traditional feature-based classification (Simulation 3). Class separation was fixed at 2.0 with equal covariance across classes; the class-relevant signal was confined to a single latent dimension, with the remaining dimensions consisting of uncorrelated noise. Error bars represent ±1 SE across 50 iterations per condition.

**Table 2:** Similarity-based classification vs. SBC and similarity-based classification vs. traditional accuracy comparisons across dimensionality (Simulation 3).

Total dims	Similarity-based classification − SBC			Similarity-based classification − Traditional		
	Diff (similarity-based classification-SBC)	*t*	*p*	Diff (similarity-based classification-Traditional)	*t*	*p*
4	0.107	24.55	<0.0001	0.003	1.25	0.215
6	0.111	23.70	<0.0001	0.003	1.13	0.264
10	0.138	31.20	<0.0001	0.009	2.99	0.004
15	0.182	31.06	<0.0001	0.008	2.38	0.021
20	0.153	27.53	<0.0001	0.006	1.51	0.138
30	0.120	18.40	<0.0001	0.015	3.31	0.002

SBC, by contrast, showed a clear decline in accuracy as dimensionality increased (from 72.06% at 4 dimensions to 63.71% at 15 dimensions, partially recovering to 68.65% at 30 dimensions), and similarity-based classification significantly outperformed SBC at every dimensionality tested (all *p*s < 0.0001; Table 2), with the gap widening as noise dimensions accumulated (mean difference at 4 dimensions = 10.70 percentage points; at 15 dimensions = 18.21 percentage points). This pattern is consistent with a structural account. Because SBC constructs features from all pairwise combinations of dimensions, the proportion of purely noise-based feature pairs grows quadratically with dimensionality. In contrast, the fraction of pairs involving the single signal dimension grows only linearly. Similarity-based and traditional feature-based representations, which retain direct access to the signal dimension regardless of the number of noise dimensions, were comparatively robust to this form of dimensional noise. As shown in the classifier-robustness analysis below, however, the magnitude of this dimensionality-related disadvantage was substantially attenuated under a nonlinear decision function.

### 3.5 Robustness to nonlinear classification

To assess whether the simulation results depended on the use of a linear decision rule, all three simulations were repeated using an RBF-kernel SVM under matched cross-validation partitions (Supplementary Figure S2). The location-based pattern in Simulation 1 was largely preserved: at a separation of 2.0, classification accuracy was 0.883 for the feature-based representation, 0.782 for SBC, and 0.887 for the similarity-based representation. In Simulation 2, however, the SBC advantage at low separation largely disappeared. At zero separation, RBF-SVM accuracy was 0.838 for the feature-based representation, 0.808 for SBC, and 0.846 for the similarity-based representation, indicating that nonlinear classification could recover dispersion-related information from the original feature and similarity-based representations. In Simulation 3, the pronounced dimensionality-related decline of SBC observed with the linear SVM was not reproduced under the RBF classifier; SBC accuracy was 0.685 at 4 dimensions, 0.756 at 15 dimensions, and 0.762 at 30 dimensions. Thus, some representational effects were robust to classifier choice, whereas others depended on the flexibility of the decision function.

## 4 Empirical application

Together, the simulations indicate that the relative effectiveness of the three representations depends not only on the statistical structure carrying class-relevant information, but also on the classifier used to read out that structure. To examine whether these patterns extend beyond controlled simulation conditions, we next applied the same three classification procedures to two empirical datasets involving affective ratings of sensory stimuli, in which the underlying statistical structure of class-relevant information is unknown and must be inferred from the data itself. Because these analyses re-used previously published datasets, we summarize here the aspects of each experiment necessary to define the classification problem and representational transformations; complete stimulus inventories and experimental procedures are available in the original reports. The two datasets were selected because they met the methodological requirements for a direct comparison of the three representations: participant-level multivariate rating profiles were available, stimuli had predefined category labels suitable for classification, and the same observations could be transformed into feature-based, intra-stimulus relational, and inter-stimulus similarity-based representations without changing the underlying measurements. They also provided complementary empirical settings that differed in sensory modality, rating dimensionality, and number of target classes. The datasets were therefore intended as methodologically suitable empirical demonstrations rather than as a representative sample of studies using multidimensional scaling.

### 4.1 Taste and music affect ratings

As a first empirical test, we applied the classification procedures to the dataset of Park and Kim (2024), which included 28 participants and 16 stimuli: eight gustatory and eight musical stimuli. Within each modality, two stimuli represented each of four target categories (sweet, bitter, sour, and salty). Participants rated each stimulus using taste and affect scales, together with liking and familiarity, yielding the 18-dimensional rating profiles used in the present analyses. Feature-based classification used the original 18 ratings, SBC transformed each profile into 153 pairwise absolute-difference features, and similarity-based classification represented the same observations using two-dimensional MDS coordinates derived from Euclidean distances among the rating profiles. Classification targeted the four taste categories separately within the gustatory and musical modalities and was evaluated using leave-one-participant-out cross-validation.

Classification performance differed across both representation and modality. For the taste stimuli, accuracy was 0.7902 for the feature-based representation, 0.7634 for SBC, and 0.4509 for the similarity-based representation. For the music stimuli, accuracy was 0.7768 for the feature-based representation, 0.7098 for SBC, and 0.7321 for the similarity-based representation. Thus, similarity-based classification was markedly less accurate for taste stimuli, whereas in the music condition it preserved substantially more class-relevant information, performing below the feature-based representation but slightly above SBC. This modality-dependent pattern suggests that the usefulness of an inter-stimulus embedding depends on the extent to which the inferred similarity geometry corresponds to the target class structure.

### 4.2 Autonomous sensory meridian response (ASMR) ratings

As a second empirical test, we applied the same three representational approaches to data from Study 2 of Kim and Kim (2022), which included 33 participants and 60 ASMR stimuli: 30 auditory and 30 audiovisual videos. Within each modality, 10 stimuli belonged to each of three predefined valence categories (positive, neutral, and negative). Participants did not assign these category labels; rather, they rated their affective responses to each stimulus on 10 adjective scales using five-point response scales, and the predefined valence categories served as the classification labels.

For each observation, feature-based classification used the original 10-dimensional affective rating profile, SBC transformed this profile into 45 pairwise absolute-difference features, and similarity-based classification represented the same observations using two-dimensional MDS coordinates derived from Euclidean distances among the rating profiles. Classification was conducted separately for the auditory and audiovisual modalities.

Classification performance was evaluated separately within each modality using leave-one-stimulus-out cross-validation: for each participant, 29 stimuli were used for training and the remaining stimulus for testing, with each of the 30 stimuli serving once as the test observation. Leave-one-stimulus-out cross-validation was used because each participant contributed 30 stimuli within each modality, allowing every stimulus to serve once as an independent test observation while retaining the remaining 29 observations for training, rather than imposing an arbitrary k-fold partition on this relatively small within-participant stimulus set.

For auditory stimuli, average classification accuracy was 0.4222 for the feature-based representation, 0.4000 for SBC, and 0.4606 for the similarity-based representation. For audiovisual stimuli, accuracy was 0.4283 for the feature-based representation, 0.4020 for SBC, and 0.4040 for the similarity-based representation. The pattern was therefore modality-dependent. The similarity-based representation showed the highest descriptive accuracy in the auditory condition, whereas the feature-based representation performed best in the audiovisual condition, with similarity-based and SBC performance being similar. These results do not indicate a general advantage of similarity-based representation, but suggest that an inter-stimulus embedding can be informative when the similarity geometry aligns sufficiently with the target class structure.

## 5 Discussion

The present findings indicate that representational choice should be treated as a substantive modeling decision in psychological classification rather than as a routine preprocessing step. Across three simulation studies and two empirical datasets, classification performance depended systematically on the correspondence between the selected representation and the structure through which class-relevant information was organized in the data. When class separation was expressed purely through the location of stimuli in a latent space (Simulation 1), similarity-based representations preserved as much discriminative information as the original feature vectors and outperformed intra-stimulus relational (SBC) representations, whose pairwise feature differences carry relatively little signal under a purely location-based structure. Notably, similarity-based and traditional feature-based classification performed comparably in the primary linear-SVM analyses, indicating that the value of the inter-stimulus embedding in this design lies primarily in preserving the relevant geometry in a lower-dimensional form rather than in producing a general predictive advantage over the original features. When class-relevant information additionally included differences in within-class dispersion (Simulation 2), SBC became more effective at low separation under the linear SVM. However, the RBF robustness analysis largely eliminated this advantage because nonlinear classification recovered dispersion-related information directly from both the feature-based and similarity-based representations. Likewise, although SBC showed a pronounced decline as task-irrelevant dimensions accumulated under the linear SVM (Simulation 3), this dimensionality-related decline was substantially attenuated under the RBF classifier. Accordingly, the results do not identify a universally superior representation. Rather, they indicate that classification performance depends jointly on the statistical information preserved by a representation and on how readily that information can be accessed by the classifier. Representational choice and classifier flexibility should therefore be considered together when evaluating alternative approaches to psychological classification. In empirical psychological data, neither the degree of class separation nor the homogeneity of within-class covariance is typically known *a priori*. Simulations 1 and 2 should therefore be interpreted as controlled boundary conditions rather than as literal models of psychological categories: Simulation 1 isolates mean-location information under equal covariance, whereas Simulation 2 illustrates how the relative performance of the representations changes when dispersion also carries class-relevant information. Accordingly, equal covariance should not be treated as a default assumption in applied settings. When the underlying structure is uncertain, as it generally is in empirical data, the relative usefulness of alternative representations must be evaluated empirically rather than inferred from a single generative condition.

The distinction between intra- and inter-stimulus representation also has broader implications for quantitative psychological research. Intra-stimulus approaches, such as SBC, represent an observation through relationships among its features or rating dimensions. In contrast, inter-stimulus approaches represent an observation through its position relative to other observations in a shared space. These representations address different questions: the former characterizes the internal organization of a psychological profile, whereas the latter characterizes its location within a broader relational geometry. This distinction connects the present study with several relational traditions in psychology, including multidimensional scaling, representational similarity analysis, network psychometrics, and perceptual modeling (Torgerson, 1958; Kruskal and Wish, 1978; Kriegeskorte et al., 2008; Diedrichsen and Kriegeskorte, 2017; Epskamp et al., 2018). Across these traditions, psychologically meaningful information is not assumed to reside exclusively in isolated scores; it may also be expressed through patterns of dependence, similarity, or relative position. The present results extend this relational perspective to classification by showing that the level at which relationships are encoded can alter the information available to a classifier. A related distinction can arise in cognitive neuroscience, where multivariate neural activity patterns may be analyzed either as feature patterns or through their pairwise representational similarities (e.g., Noda et al., 2024). The present distinction between feature-level and relational representations may therefore be conceptually relevant to neural representational analyses as well. However, because the present simulations and empirical applications were based on behavioral and psychophysical data, the relative performance of these approaches should not be assumed to generalize directly to neural data.

The empirical analyses illustrate why representational alignment must be evaluated rather than assumed. This issue may be particularly important when experimentally imposed category labels do not closely correspond to how participants actually perceive the organization of the stimuli. In such cases, the correspondence between predefined categories and empirical similarity geometry can itself provide information about whether the assumed class structure reflects psychologically meaningful distinctions. In the ASMR data, the similarity-based representation produced the highest descriptive accuracy in the auditory condition, suggesting that valence-related information was at least partly organized through continuous relationships among stimuli. However, this advantage was not observed in the audiovisual condition, where the feature-based representation performed best. A similarly modality-dependent pattern emerged in the taste and music data. Similarity-based classification was markedly less accurate for taste stimuli, whereas for music it preserved substantially more discriminative information, performing below the feature-based representation but slightly above SBC. These contrasting results are more informative as evidence of boundary conditions than as evidence for the general superiority of any single representation. They suggest that an inter-stimulus embedding can retain useful discriminative information when similarity geometry and class structure correspond, but can lose such information when that correspondence is weak.

Although the three approaches were treated separately here to permit a controlled comparison, they need not be mutually exclusive. In representational similarity analysis, theoretically specified or measured stimulus features can be expressed as model dissimilarity structures and related to an empirical similarity space, allowing feature-level information and inter-stimulus geometry to be examined within a common framework (Kriegeskorte et al., 2008; Nili et al., 2014; Diedrichsen and Kriegeskorte, 2017). Such hybrid approaches may be particularly useful when the goal is not only to classify stimuli but also to identify which specific features account for the organization of the observed similarity structure.

A practical implication is that representational choice should follow the hypothesized source of category-relevant information rather than be treated as a neutral preprocessing step. Feature-based representations may be most appropriate when theoretically meaningful absolute feature levels are available and expected to distinguish categories. Intra-stimulus relational representations may be preferable when categories are hypothesized to differ in the configuration of features within an observation, as emphasized by the SBC framework (Kim, 2026). Inter-stimulus representations are particularly relevant when the psychological hypothesis concerns how stimuli are positioned relative to one another within a shared perceptual or representational space (Kriegeskorte and Kievit, 2013; Diedrichsen and Kriegeskorte, 2017). Although examined here only with affective and sensory ratings, the same representational question may arise in other multivariate psychological applications, such as perceptual or cognitive decoding and clinical prediction, where observations are represented by multiple measured features or by similarities among observations (Pereira et al., 2009; Dwyer et al., 2018; Yarkoni and Westfall, 2017). Whether the relative performance patterns observed here generalize to those domains remains an empirical question. In practice, comparison across plausible representations serves not only to optimize prediction but also to clarify how psychologically meaningful distinctions are organized in a dataset (Yarkoni and Westfall, 2017). Researchers working with high-dimensional rating batteries should also be aware that intra-stimulus relational representations may become less reliable as the number of measured dimensions grows relative to sample size. In contrast, inter-stimulus and feature-based representations were comparatively robust to this form of dimensional noise in the present simulations.

Several limitations should qualify these conclusions. First, statistical inference was available for the simulation comparisons, which were evaluated using paired tests across repeated iterations, but not for the empirical comparisons, which involved a single application per dataset and should therefore be interpreted as descriptive rather than inferential evidence. Future work should incorporate resampling-based inference (e.g., bootstrap confidence intervals or permutation tests) to evaluate the reliability of representational differences in empirical data formally. Second, although the three simulations examined location-based separation, dispersion differences, and dimensionality independently, these factors were not fully crossed, and their joint influence on representational performance remains untested. Future simulations should manipulate these factors in a factorial design to examine how representations perform under combinations of location, dispersion, and dimensionality effects. Third, the empirical applications were restricted to affective and sensory ratings. Although the representational framework is potentially relevant to cognitive, clinical, personality, and behavioral data, its usefulness in those domains remains to be tested directly. Fourth, the similarity-based approach depends on the quality, dimensionality, and stability of the dissimilarity structure. Similarity estimates may be noisy, sparse, or heterogeneous across individuals, and a single group-level embedding may conceal meaningful individual differences. Fifth, although the primary analyses used a linear SVM and were supplemented by an RBF-kernel robustness analysis, only two decision functions were examined and no hyperparameter optimization was performed. Other classifier families and tuning strategies may produce additional representation–classifier interactions. The present similarity-based implementation was also restricted to a two-dimensional MDS solution; alternative dimensionality-selection procedures, individual-differences scaling (e.g., Carroll and Chang, 1970), or other embedding methods may yield different results. In conclusion, the present study shows that classification outcomes in psychology depend not only on the learning algorithm but also on the representation of psychological information. Feature-based, intra-stimulus, and inter-stimulus representations preserve different aspects of the data and should therefore be selected according to substantive assumptions about where category-relevant information resides. Treating representation as an explicit methodological and theoretical choice may improve both predictive performance and the interpretation of psychological classification models.

## Data Availability

The datasets presented in this study can be found in online repositories. The names of the repository/repositories and accession number(s) can be found at the data and materials supporting the findings of this study are openly available in the Open Science Framework (OSF) repository at https://osf.io/e5vxd/. The repository includes the full simulation pipeline, including code for data generation, feature construction, and visualization. Empirical datasets used in this study are available from the original published sources cited in the manuscript.

## Supplementary material

Supplementary Figure S1 Representative examples of the three representational approaches across simulations. Rows correspond to Simulation 1 (equal covariance, separation = 2.0), Simulation 2 (unequal covariance, separation = 0.0), and Simulation 3 (15 dimensions, separation = 2.0), respectively. Columns show the original feature-based representation, the SBC representation constructed from pairwise absolute differences among feature dimensions, and the two-dimensional similarity-based representation obtained from inter-stimulus Euclidean distances using multidimensional scaling. In the heatmaps, stimuli from Class A and Class B are shown in the upper and lower halves, respectively. In the MDS plots, circles and triangles denote the two classes. For each condition, the displayed realization was selected as the iteration whose three-method classification-accuracy vector was closest to the condition-wise mean across iterations.

Supplementary Figure S2 Robustness of classification performance to classifier choice across the three simulations. Rows correspond to Simulations 1–3, and columns correspond to linear and RBF SVM classifiers. For Simulations 1 and 2, mean classification accuracy (± standard error) is plotted as a function of class separation; for Simulation 3, accuracy is plotted as a function of total dimensionality. Curves show the feature-based, SBC, and similarity-based representations. The same 10-fold cross-validation partition was used across representations and classifiers within each simulated dataset. Error bars represent ±1 SE across 100 iterations for Simulations 1 and 2 and 50 iterations for Simulation 3.

## References

[ref9001] Carroll J. D. Chang J. J. (1970). Analysis of individual differences in multidimensional scaling via an N-way generalization of “Eckart–young” decomposition. Psychometrika 35, 283–319. doi: 10.1007/bf02310791

[ref2] Diedrichsen J. Kriegeskorte N. (2017). Representational models: a common framework for understanding encoding, pattern-component, and representational-similarity analysis. PLoS Comput. Biol. 13:e1005508. doi: 10.1371/journal.pcbi.1005508, 28437426 PMC5421820

[ref3] Dwyer D. B. Falkai P. Koutsouleris N. (2018). Machine learning approaches for clinical psychology and psychiatry. Annu. Rev. Clin. Psychol. 14, 91–118. doi: 10.1146/annurev-clinpsy-032816-045037, 29401044

[ref4] Epskamp S. Borsboom D. Fried E. I. (2018). Estimating psychological networks and their accuracy: a tutorial paper. Behav. Res. Methods 50, 195–212. doi: 10.3758/s13428-017-0862-1, 28342071 PMC5809547

[ref5] Friedman H. H. Amoo T. (1999). Rating the rating scales. J. Mark. Manag. 9, 114–123. doi: 10.63963/001c.150748

[ref6] Heine S. J. Lehman D. R. Peng K. Greenholtz J. (2002). What’s wrong with cross-cultural comparisons of subjective Likert scales? The reference-group effect. J. Pers. Soc. Psychol. 82, 903–918. doi: 10.1037/0022-3514.82.6.903, 12051579

[ref7] Kim J. (2026). Structure-based classification approach. Appl. Psychol. Meas. 50, 49–54. doi: 10.1177/01466216251360544, 40678359 PMC12264251

[ref8] Kim H. Kim J. (2022). Affective responses to ASMR using multidimensional scaling and classification. Korean Soc. Emot. Sensib. 25, 47–62. doi: 10.14695/kjsos.2022.25.3.47

[ref9] Kriegeskorte N. Kievit R. A. (2013). Representational geometry: integrating cognition, computation, and the brain. Trends Cogn. Sci. 17, 401–412. doi: 10.1016/j.tics.2013.06.007, 23876494 PMC3730178

[ref10] Kriegeskorte N. Mur M. Bandettini P. (2008). Representational similarity analysis—connecting the branches of systems neuroscience. Front. Syst. Neurosci. 2:4. doi: 10.3389/neuro.06.004.2008 PMC2605405 19104670

[ref11] Kruskal J. B. Wish M. (1978). Multidimensional Scaling. Beverly Hills, CA: Sage Publications.

[ref12] Nili H. Wingfield C. Walther A. Su L. Marslen-Wilson W. Kriegeskorte N. (2014). A toolbox for representational similarity analysis. PLoS Comput. Biol. 10:e1003553. doi: 10.1371/journal.pcbi.1003553, 24743308 PMC3990488

[ref13] Noda T. Aschauer D. F. Chambers A. R. Seiler J. P. H. Rumpel S. (2024). Representational maps in the brain: concepts, approaches, and applications. Front. Cell. Neurosci. 18:1366200. doi: 10.3389/fncel.2024.1366200, 38584779 PMC10995314

[ref14] Park C. Kim J. (2024). Taste the music: modality-general representation of affective states derived from auditory and gustatory stimuli. Cognition 249:105830. doi: 10.1016/j.cognition.2024.105830, 38810426

[ref15] Pereira F. Mitchell T. Botvinick M. (2009). Machine learning classifiers and fMRI: a tutorial overview. NeuroImage 45, S199–S209. doi: 10.1016/j.neuroimage.2008.11.007, 19070668 PMC2892746

[ref16] Schwarz N. (1999). Self-reports: how the questions shape the answers. Am. Psychol. 54, 93–105. doi: 10.1037/0003-066x.54.2.93

[ref17] Torgerson W. S. (1958). Theory and Methods of Scaling. New York, NY: Wiley.

[ref18] Yarkoni T. Westfall J. (2017). Choosing prediction over explanation in psychology: lessons from machine learning. Perspect. Psychol. Sci. 12, 1100–1122. doi: 10.1177/1745691617693393, 28841086 PMC6603289

